# Targeted Single-cell Isolation of Spontaneously Escaping Live Melanoma Cells for Comparative Transcriptomics

**DOI:** 10.1158/2767-9764.CRC-22-0305

**Published:** 2023-08-11

**Authors:** Jacqueline L.E. Tearle, Satya N.V. Arjunan, Szun S. Tay, Feyza Colakoglu, James Cremasco, Matteo Golo, Maté Biro

**Affiliations:** 1 EMBL Australia, Single Molecule Science node, School of Biomedical Sciences, University of New South Wales, Sydney, Australia.; 2 Present address: Garvan Institute of Medical Research, Darlinghurst, New South Wales, Australia.; 3 Present address: Victor Chang Cardiac Research Institute, Darlinghurst, New South Wales, Australia.

## Abstract

Solid cancer cells escape the primary tumor mass by transitioning from an epithelial-like state to an invasive migratory state. As they escape, metastatic cancer cells employ interchangeable modes of invasion, transitioning between fibroblast-like mesenchymal movement to amoeboid migration, where cells display a rounded morphology and navigate the extracellular matrix in a protease-independent manner. However, the gene transcripts that orchestrate the switch between epithelial, mesenchymal, and amoeboid states remain incompletely mapped, mainly due to a lack of methodologies that allow the direct comparison of the transcriptomes of spontaneously invasive cancer cells in distinct migratory states. Here, we report a novel single-cell isolation technique that provides detailed three-dimensional data on melanoma growth and invasion, and enables the isolation of live, spontaneously invasive cancer cells with distinct morphologies and invasion parameters. Via the expression of a photoconvertible fluorescent protein, compact epithelial-like cells at the periphery of a melanoma mass, elongated cells in the process of leaving the mass, and rounded amoeboid cells invading away from the mass were tagged, isolated, and subjected to single-cell RNA sequencing. A total of 462 differentially expressed genes were identified, from which two candidate proteins were selected for further pharmacologic perturbation, yielding striking effects on tumor escape and invasion, in line with the predictions from the transcriptomics data. This work describes a novel, adaptable, and readily implementable method for the analysis of the earliest phases of tumor escape and metastasis, and its application to the identification of genes underpinning the invasiveness of malignant melanoma.

**Significance:**

This work describes a readily implementable method that allows for the isolation of individual live tumor cells of interest for downstream analyses, and provides the single-cell transcriptomes of melanoma cells at distinct invasive states, both of which open avenues for in-depth investigations into the transcriptional regulation of the earliest phases of metastasis.

## Introduction

Despite the emergence of ground-breaking combination therapies and immunotherapies in recent years, 5-year survival rates for highly metastatic cancers remain low (1). To identify new therapeutic targets for the prevention of metastasis, the underlying drivers of its earliest stages need to be more completely mapped. The propensity for cancer cells to migrate away from a primary tumor relies on their ability to escape the cohesive bulk tumor mass and subsequently invade through barriers and tissues of varying architecture, composition, and biochemical landscape. To do so, cancer cells must first acquire an invasive phenotype, typically via an epithelial-to-mesenchymal transition (EMT), and then employ varying modes of migration (2–5).

Individually migrating cells can employ different modes of migration that were classically divided into two categories: elongated, integrin-dependent mesenchymal migration (2), and rounded, integrin-independent amoeboid migration (3–5). Similarly to EMT, cells can switch between these two modes of migration via a mesenchymal-to-amoeboid transition (MAT) or an amoeboid-to-mesenchymal transition (AMT; refs. 6, 7). As in the case of EMT, cells can also display hybrid phenotypes ensuing partial transitions between the amoeboid and mesenchymal extremes. Amoeboid cells do not display the sheet-like lamellipodia and integrin-based crawling style characteristic of mesenchymal cells, nor do they typically secrete proteases that enzymatically digest the extracellular matrix (ECM; refs. 3–5). Instead, amoeboid cells “push and squeeze” their way through gaps in the ECM using a combination of two key mechanisms: friction-mediated and propulsion-mediated locomotion, which both rely heavily on actomyosin contractility (2, 8). Although shifts from mesenchymal to amoeboid migration have been observed in highly aggressive cancers, and migratory plasticity is associated with increased invasiveness, little is known about the mechanisms that induce MAT and AMT in cancer (6, 7).

Numerous studies have attempted to isolate cells before and after transitions to identify changes in gene expression underpinning metastatic spread (6, 9–14). However, these studies were limited in that (i) they did not capture early or spontaneous tumor escape events (without the use of exogenous inducing agents), (ii) they did not allow for the specific analysis of cells displaying different migratory phenotypes, or (iii) they did not allow for the sorting of live single cells of a defined phenotype. This is in part due to the lack of a suitable methodology that enables the effective isolation of single cells pre- and post-transition at distinct stages of spontaneous tumour escape.

Here, we present a novel single-cell isolation method, which allows for the targeted isolation of single cells spontaneously escaping a melanoma spheroid mass and of a specific phenotype for downstream transcriptomics analysis. We employ the method to isolate and compare the transcriptomes of individual metastatic melanoma cells in three distinct states: pre-invasive “epithelial-like” cells at the edge of the primary tumor mass; elongated and actively escaping cells still attached to primary tumor mass; and rounded cells invading away from the tumor in an amoeboid-like manner.

## Materials and Methods

### Cell Culture

The WM983c melanoma cell line (gift from N. Haass, University of Queensland, Queensland, Australia, RRID:CVCL_A338) was transduced with lentivirus containing the photoconvertible fluorescent protein Dendra2 (15) [cloned into pRRLSIN-eGFP (RRID:Addgene_176015), gift from I. Alexander, CMRI, via restriction sites *BamHI* and *SalI* to replace EGFP] produced in HEK293T cells (ATCC CRL-3216, RRID:CVCL_0063) cotransfected with psPAX2 (RRID:Addgene_12260) and pVSVG (RRID:Addgene_85140). Transduced cells were single-cell sorted into 96-well plates on a BD FACS Aria III flow cytometer/cell sorter (BD Biosciences), from which the clonal WM983c-D2 cell line was expanded. Single-cell clonal populations were used in this study to minimize genomic heterogeneity between cells and to capture the most fundamental changes in gene expression driving tumor escape and amoeboid migration. For all experiments presented, cells were thawed at passage 50–60 and subjected to at least two passages before spheroid generation. Cells were kept in a 5% CO_2_ humidified incubator at 37°C and were split every 48–72 hours at 80% confluency. The WM983c cell line used in this study was most recently authenticated by CellBank Australia on June 4, 2021, and tested negative for *Mycoplasma* enzyme via MycoAlert *Mycoplasma* Detection Kit (Lonza) on September 3, 2020. Most experiments were conducted between 2020 and 2021.

### Generation of Three-dimensional Spheroids in Collagen Matrix

**FIGURE 1:**
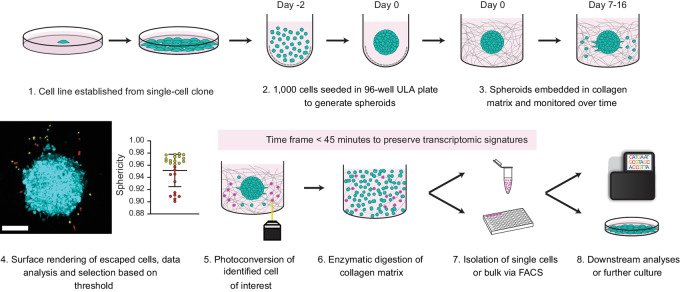
Methodologic pipeline for single-cell isolation of live escaping tumor cells. **1,** A clonal population of tumor cells are expanded from a single cell. **2,** Photoconvertible cells are seeded in ULA plates to generate spheroids. **3,** Spheroids are embedded within a 3D collagen matrix and imaged over time by light microscopy. **4,** Image analysis and extraction of quantitative metrics can be used to select cells of interest. **5,** Single cells of interest are photoconverted using a 405 nm laser. **6,** Collagen matrix is enzymatically digested to provide a solution of suspended single cells. **7,** Cells are sorted via FACS. **8,** Cells can be further cultured or immediately analyzed.

A total of 10^3^ WM983c melanoma cells were seeded in 50 μL of culture medium in wells of a 96-well ultra-low attachment (ULA) plate (CLS7007, Corning Costar) and incubated at 37°C in a 5% CO_2_ humidified incubator to form spheroids (Fig. 1). After 48 hours, spheroids were implanted into collagen matrix in individual wells of 96-well glass-bottomed imaging plates (Sensoplate microplate M4187, Greiner Bio-One). All collagen matrix reagents were kept on ice and mixtures prepared on ice to prevent premature collagen polymerization. Collagen matrices were prepared using the following ratio of reagents and scaled up as required: 10 μL 10x PBS, 1.15 μL NaOH, 50 μL complete culture medium, 50 μL rat tail collagen type I (4 mg/mL stock, RatCol #5153, Advanced Biomatrix). Before spheroid implantation, a 30 μL layer of collagen mixture containing no cells was applied to the bottom of each well and transferred to a 37°C 5% CO_2_ humidified incubator for 3 minutes to partially polymerize. This is to ensure that the implanted spheroids do not sink to the bottom of the plate during matrix polymerization. Then, a 50 μL collagen layer was applied, containing a single spheroid within the culture medium, taken from the ULA plates incubated 48 hours previously. Samples were returned to 37°C for a further 15 minutes to allow the collagen to completely polymerize before the addition of 200 μL prewarmed complete bathing medium. Spheroids were kept in a 37°C 5% CO_2_ humidified incubator for the duration of the study.

### Three-dimensional Imaging and Photoconversion

Spheroids were imaged and z-stacks acquired every 1–3 days using a 10x objective on a Leica DLS SP8 confocal imaging system (Leica Microsystems, RRID:SCR_018169; Supplementary Fig. S1). Dendra2^green^ fluorescence was acquired by excitation at 488 nm and detection at 500–550 nm. To observe photoconverted cells, Dendra2^red^ fluorescence was acquired by excitation at 561 nm and detection at 570–650 nm. Three-dimensional (3D) images were visualized and surface rendered using Imaris software (Bitplane AG, RRID:SCR_007370). Individual cells were photoconverted (Fig. 1; Supplementary Fig. S1A–S1F) using 10% confocal zoom and the region of interest function to focus the 405 nm laser on specific cells for 10–20 seconds at 30% laser power (nominal power: 2 mW). Only single cells at the periphery and cells without obstruction from other cells were photoconverted to ensure no off-target photoconversion (Supplementary Fig. S1B). While escaping and amoeboid cells were photoconverted one cell at a time, groups of epithelial cells were photoconverted together due to their compact packing. To conserve the transcriptomics signature of isolated cells, no more than 45 minutes were allowed to lapse between cell photoconversion and sorting/lysis. This is based on the optimized minimum time needed to obtain quality imaging data, and that “fast” decaying mRNA transcripts exhibit a detectable half-life of <2 hours (16), and transcription of immediate early genes can be detected within 30 minutes (17). Of note, cells were collected from a range of days to ensure any enriched genes were phenotype-specific, rather than a manifestation of spheroid age (Supplementary Table S1).

### Quantification of Spheroid Growth and Invasion Data

3D images were surface rendered, visualized, and analyzed using Imaris software version 9.2.1 (Bitplane, RRID:SCR_007370). Because of limitations of single photon imaging, where laser light cannot effectively penetrate through dense tissues, it is not possible to obtain emission data from cells located behind the dense spheroid. Therefore, 3D images were cropped at a Z-depth corresponding to the widest point of the spheroid for all further analysis (Supplementary Fig. S1C). To obtain data on spheroid size, automatic isolines were fitted to generate an ellipse around the spheroid periphery at the maximum Z-depth “z” as shown in Supplementary Fig. S1C and S1D. Data can then be exported from the Imaris software to provide the radius at the widest point “a” and narrowest point “b” of the ellipse (Supplementary Fig. S1D). Values *a*, *b*, and *z* were then averaged to get a mean radius *r*. The mean radius value was then used to calculate an estimated total spheroid volume as per the equation below:



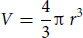



Where *V* is estimated spheroid volume in μm^3^ and *r* is the mean radius in μm. Surface area of the imaged hemispheroid was calculated using the surface area of a spherical cap formula:



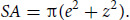



Where *SA* is the surface area of the spherical cap in μm^2^, *e* is the mean ellipsoid axis calculated from ellipsoid axes *a* and *b* in μm, and *z* is the imaging depth in μm. The number of escaped cells per day was normalized to the hemispheroid surface area, to derive cells per μm^2^ as a measure of invasion.

**FIGURE 2:**
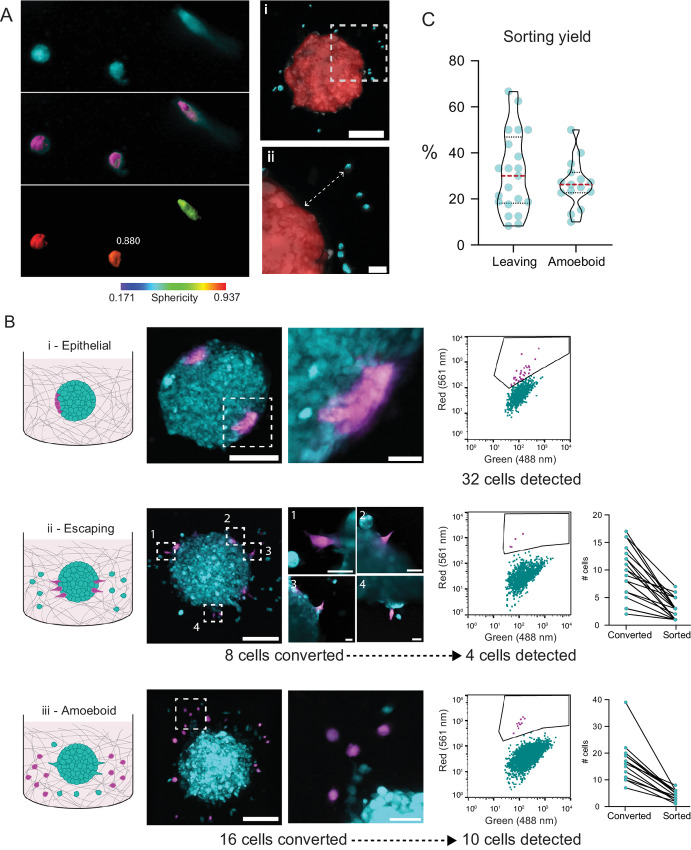
Single-cell tagging and isolation based on morphologic and positional data. **A,** Sphericity and distance to spheroid values are generated for each invading cell surrounding the spheroid. Left panel depicts maximum intensity projections for three invading cells (Fluo) of different sphericities, following cell surface segmentation (Fluo + Surf), and following surface pseudocoloring according to sphericity value (Surf + Pseudo). Right panels illustrate the smoothed spheroid surface (red) computed to calculate the nearest distance (dashed arrow) to the spheroid edge from individual cells. **B,** Left: The three photoconverted cell types “Epithelial,” “Escaping,” and “Amoeboid.” Middle: Representative images of the photoconversion of the three cell types and corresponding flow cytometry scatter plots of Dendra2 red versus green fluorescence obtained during single-cell sorting. Right: The number of cells photoconverted and recovered via single-cell sorting for escaping and amoeboid cells. **C,** Sorting yields expressed as a percentage of photoconverted cells for escaping and amoeboid cells.

To obtain individual escaped cell metrics, cropped 3D images were surface rendered using the built-in surfaces function within the Imaris software with the smooth surfaces option disabled. The surface corresponding to the spheroid mass was then removed, leaving behind only surfaces corresponding to escaped cells (Supplementary Fig. S1E). As mentioned above, due to cropping of the 3D image at the widest point of the spheroid, only cells found within the specified Z-depth were analyzed as depicted in Supplementary Fig. S1F. Cell metrics for individual cell surfaces were calculated in Imaris and then exported for further analysis (Supplementary Fig. S1G and S1H). Figure 2A shows how surfaces are generated from 3D fluorescence data, with individual sphericity values for three cells provided as an example. Sphericity defines how spherical a surface is on a scale of 0 to 1, where 1 would be perfectly spherical, and is defined in the equation below:



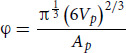



Where Ψ is sphericity, *V*_p_ is cell volume, and *A*_p_ is cell surface area. To obtain information on how far each cell had travelled from the edge of the growing spheroid, we generated a smoothed surface around the spheroid to use as a reference (Fig. 2Ai and Aii). A smoothed surface was used to ensure the distance from spheroid measurements reflected the distance a cell had travelled from the bulk spheroid core, and not from any elongated invasive cells still attached to the spheroid periphery. The distance in μm from every cell to the smoothed spheroid surface was exported into a single spreadsheet for downstream analysis.

### Single-cell Isolation

Bathing medium was aspirated and replaced with 200 μL warm culture medium containing 0.2 mg mL^−1^ collagenase from *Clostridium histolyticum* (Sigma-Aldrich, #C5138) for enzymatic digestion of the collagen matrix for 10 minutes at 37°C and 5% CO_2_ (Fig. 1). After 10 minutes, complete dissociation of the matrix was assisted by trituration before returning to the incubator for a further 10 minutes. Once completely dissociated, samples were again triturated to create a single-cell suspension, transferred to 0.5 mL microcentrifuge tubes and centrifuged for 5 minutes at 300 × *g*. Supernatants were discarded and pellets were washed twice with cold FACS wash (2 mmol/L ethylenediaminetetraacetic acid [EDTA] and 2% FCS in 1x PBS). Pellets were then resuspended in 200 μL FACS wash and passed through 35 μm cell strainers (Corning, Falcon #352235) into 5 mL FACS tubes on ice.

Cell sorting was performed using the BD Influx cell sorter (BD Biosciences) equipped with 488 and 561 nm lasers, with the 488 nm laser set to 20% laser power to avoid photoswitching cells during sorting. Single cells were identified depending on side scatter (SSC) versus polarized forward scatter (FSC S-Pol) and doublet exclusion (SSC area vs. SSC and trigger pulse width vs. FSC S-Pol). Spheroids with no photoconverted cells were processed alongside photoconverted samples and used as negative controls for gating. Single Dendra2^red^ cells (488^+^/561^+^) were gated and sorted into 96-well PCR plates containing 4 μL lysis buffer as described in ref. 18. Immediately after sorting, PCR plates were centrifuged at 300 × *g* at 4°C for 2 minutes before being flash frozen on dry ice. Plates were then stored at −80°C until used for library preparation and single-cell transcriptomics.

### Single-cell Transcriptomics and Generation of Count Files (performed by Admera Health)

cDNA synthesis was performed on single cells using the SMART-Seq v4 Ultra Low Input RNA Kit for Sequencing as per manufacturer's instructions. Library preparation was performed using the Nextera XT kit and libraries were sequenced on an Illumina sequencing platform with a read length of 2 × 150 bp, 5M reads. FastQC (version v0.11.8, RRID:SCR_014583) was applied to check the quality of raw reads. Trimmomatic (version v0.38, RRID:SCR_011848) was applied to cut adaptors and trim low-quality bases with default setting. STAR Aligner (version 2.7.1a) was used to align reads. Picard tools (version 2.20.4) was applied to mark duplicates of mapping. StringTie (version 2.0.4) was used to assemble RNA sequencing (RNA-seq) alignments into potential transcripts. FeatureCounts (version 1.6.0) was used to count mapped reads for genomic features such as genes, exons, promoters, gene bodies, genomic bins, and chromosomal locations, generating count files which were used for subsequent analyses.

### Differential Expression, Clustering Analyses, and Gene Set Enrichment Analysis

Differential expression (DE) analysis was performed with the DESeq2 (19) software (RRID:SCR_011848) using default options to generate lists of DE genes for three pairwise comparisons: escaping versus epithelial, amoeboid versus escaping, and amoeboid versus epithelial. Variance stabilizing transformation (VST) was performed on raw count files before clustering. Unsupervised hierarchical clustering was performed with the publicly available and established hierarchical clustering method “scipy.cluster.hierarchy.linkage” (20), using the Ward variance minimization algorithm and the Euclidean distance metric to cluster cells based on expression levels. DESeq2 analysis on 44 cells yielded 230 DE genes and clustering analysis on 44 cells identified one outlier cell (E-44) as well as a cluster of cells with comparatively low gene expression (Supplementary Fig. S2). To determine whether the low gene expression within these cells was biologically relevant, or merely as a result of poor sequencing quality, we analyzed the raw counts of 22 common housekeeping genes (21, 22) for 43 cells (following the removal of E-44). This identified the same clear outliers with consistently low expression of housekeeping genes. A threshold was therefore applied to exclude from further analysis any cells where more than 30% of housekeeping genes had <10 raw counts, which led to the exclusion of a further eight cells (A-59, A-60, A-62, E-13, Es-30, Es-36, Es-37, and Es-55) as indicated in Supplementary Table S2, to give a final dataset of 35 cells. Subsequent DESeq2 analysis on 35 cells identified 462 DE genes. Gene set enrichment analysis (GSEA, RRID:SCR_003199) was carried out on VST normalized counts using the GSEA software v4.1.0 as per the relevant documentation (23, 24). Uniform manifold approximation and projection (UMAP) analysis was carried out on VST-normalized data using the nearest neighbors and UMAP tools within the publicly available SCANPY package (25).

### Spheroid Assays with Pharmacologic Treatments

3D spheroids were generated from WM983c cells as described earlier. Spheroids were implanted on day −1 and left to recover overnight. On day 0, spheroids were imaged before the replacement of bathing medium containing 15 μmol/L naltrindole hydrochloride (Sigma-Aldrich, #N115) or 1 mmol/L sulfasalazine (SSZ; Sigma-Aldrich, #S0883). Vehicle conditions for naltrindole and SSZ experiments were 3 μL H_2_O or 2 μL DMSO per 200 μL bathing medium, respectively. Spheroids were then imaged daily for 7 to 10 days.

### Western Blot Analysis

At least 1 million cells were pelleted by centrifugation for 5 minutes at 1,200 rpm and washed twice with ice-cold PBS. Cell pellets were then resuspended in 50–200 μL RIPA lysis buffer (#89900 Thermo Fisher Scientific) with 1:100 protease inhibitor cocktail (#78429, Thermo Fisher Scientific) and left on ice for at least 30 minutes, vortexing every 10 minutes. Samples were then centrifuged at 12,000 rpm for 15 minutes at 4°C and protein-containing supernatants were isolated and stored at −20°C until use. Protein concentrations were determined by bicinchoninic acid (BCA) assay using the Pierce BCA protein assay kit (23227, Thermo Fisher Scientific) as per manufacturer's instructions. A total of 20 μg of protein was combined with 7.5 μL NuPAGE Sample Buffer (#NP0007, Thermo Fisher Scientific) and 3 μL NuPAGE Sample Reducing Agent (#NP0004, Thermo Fisher Scientific) in a total of 30 μL and ran at 100 V for 1 hour on a NuPAGE 10% Bis-Tris 1 mm Protein Gel (#NP0301BOX, Invitrogen) as per manufacturer's instructions. Protein was transferred to membrane using an iBlot 2 Dry Blotting System (Invitrogen) with iBlot 2 NC ministacks (IB23003, Invitrogen) as per manufacturer's instructions.

Membranes were washed twice in 1x TBS with 0.1% Tween detergent (TBST) and then incubated while rocking at room temperature in 5% low-fat milk powder dissolved in TBST for 1 hour. Membranes were then incubated with primary antibody in 5% milk powder-TBST (OPRD1 rabbit polyclonal #AB1560 RRID:AB90778, MilliporeSigma, 1:1,000; SLC19A1 rabbit polyclonal #AB272354 RRID:AB_2885134, Abcam, 1:1,000; Tubulin rat monoclonal #AB6160 RRID:AB_205328, Abcam, 1:5,000) overnight on a rocker for at least 16 hours at 4°C. Membranes were then washed four times with TBST before the addition of species-appropriate secondary antibody in 5% milk powder-TBST and incubation on a rocker for 1 hour at room temperature (anti-rat HRP raised in goat, polyclonal #AB160047, Abcam, 1:10,000; Anti-rabbit HRP raised in goat, polyclonal #AB97051 RRID:AB_10679369, Abcam, 1:5,000).

### Immunofluorescence Staining of Spheroids

Spheroids were embedded and cultured as described above. On day 10, spheroids were fixed and permeabilized using the BD Cytofix/Cytoperm kit (#AB 2869008, BD) as per manufacturer's instructions. Spheroids were incubated for 24 hours at 4°C in the absence of light with SLC19A1 primary antibody (detailed above) at 1:200 in wash buffer supplied in the Cytofix/Cytoperm kit. Spheroids were then washed twice in wash buffer in the dark before secondary incubation for 24 hours with anti-rabbit A647 secondary antibody at 1:200 in wash buffer at 4°C as before. Spheroids were then washed twice and bathed in wash buffer for imaging. Imaging was carried out as described above on a Leica SP8 imaging system (Supplementary Fig. S3). 3D image analysis software (Imaris, Bitplane) was used to generate surfaces for escaped cells (cyan surfaces, Supplementary Fig. S3C), leaving cells (magenta surfaces, Supplementary Fig. S3C), and one central surface comprising the cohesive bulk spheroid to represent the epithelial cell population (yellow surface, Supplementary Fig. S3C). Mean fluorescence intensity for respective channels for each surface was used to quantify protein expression.

### Statistical Analysis

All statistical analyses were performed in Prism software version 9 (Graphpad, RRID:SCR_000306). Statistical significance tests were Wilcoxon rank-sum or Kruskal–Wallis H as appropriate and indicated, with significance reported in figures by asterisks as follows: *P* > 0.05, not significant (not shown); *, *P* ≤ 0.05; **, *P* ≤ 0.01; ***; *P* ≤ 0.001; ****; *P* ≤ 0.0001. All analyses are nonsignificant unless stated otherwise.

### Data Availability

The transcriptomics data generated in this study are publicly available in the EMBL-EBI ArrayExpress database (http://www.ebi.ac.uk/arrayexpress) at under accession number E-MTAB-11722.

## Results

### The Photoconvertible 3D Spheroid Method Allows for the Targeted Isolation of Individual Live Escaping Cells

We sought to design a workflow capable of analyzing tumor growth and invasion in 3D, as well as enabling the isolation of live single cells based on phenotype for further downstream analysis (Fig. 1). The human metastatic melanoma cell line WM983c was transduced with Dendra2 (15), and a clonal, Dendra2-positive cell population was expanded from a single cell (hereafter WM983c-D2). Spheroids were generated via seeding in ULA plates, and after 48 hours, individual spheroids were embedded within 3D collagen matrices for image analysis. A total of 46 spheroids from five independent experiments were confocally imaged in three dimensions every 24–72 hours to collect data on spheroid volume and invasive cell characteristics such as morphological parameters and distance from spheroid edge (Fig. 2A; Supplementary Fig. S1G). Spheroids steadily increased in volume (Supplementary Fig. S1G i) and invasiveness (Supplementary Fig. S1G ii) over time. Mean escaped cell distance from the spheroid core increased over time as cells invaded further away into the surrounding ECM (Supplementary Fig. S1G iii), and escaped cells maintained a high sphericity index between 0.8 and 0.9 throughout the experiment (Supplementary Fig. S1G iv). All cell and spheroid metrics were obtained from 3D data.

Spheroids grown from WM983c-D2 cells generate cells of three distinct phenotypes: (i) non-invading cells organized in a compact manner within the spheroid surface with no visible cell protrusions, hereafter termed “epithelial”; (ii) cells still in contact with the spheroid periphery that have commenced tumor escape, exhibiting cellular protrusions and an elongated shape, hereafter termed “escaping”; (iii) singly migrating cells detached from the spheroid and exhibiting a rounded phenotype consistent with amoeboid cell migration, hereafter termed “amoeboid” (Fig. 2B, left). Target cells within spheroids were then photoconverted and processed for single-cell isolation on days 6–16 post-implantation, depending on the type of cell isolated (epithelial, escaping, or amoeboid; Fig. 2B). To conserve the transcriptomics signature of isolated cells, the time taken between photoconversion and cell lysis was limited to less than 45 minutes for all samples (16, 17).

A total of 48 epithelial, 56 escaping, and 49 amoeboid cells were isolated from 12, 21, and 13 individually processed spheroids, respectively (Supplementary Figs. S4–S6). On average, 31% of photoconverted escaping cells and 27% of photoconverted amoeboid cells were successfully isolated and recovered by FACS (Fig. 2B and C). Because of the compact nature of epithelial cells, it was not possible to precisely determine the number of cells photoconverted to generate an exact yield, but we estimate it to be similar to the isolation efficiency of escaping and amoeboid cells. As expected, due to their characteristic rounded morphology (2), amoeboid cells exhibited a higher sphericity value than escaping cells (Supplementary Fig. S7A). The mean distance of amoeboid cells from the spheroid edge was 114 μm (Supplementary Fig. S7B), though cells from a wide range of invasion distances were obtained. Following reverse transcription, seven cells were rejected because of low cDNA concentration (Supplementary Fig. S7C). Following sequencing, 102 cells were rejected because of inadequate mapping rates to the reference genome (Supplementary Fig. S7D), and mapping rate correlated with cDNA concentration (Supplementary Fig. S7E). In total, 44 cells were put forward for further analysis (Supplementary Fig. S7F): 12 epithelial cells, 16 escaping cells, and 16 amoeboid cells.

### Single-cell Sequencing Reveals Distinct Transcriptomics Signatures as Cells Progress from Epithelial to Escaping to Amoeboid States

**FIGURE 3:**
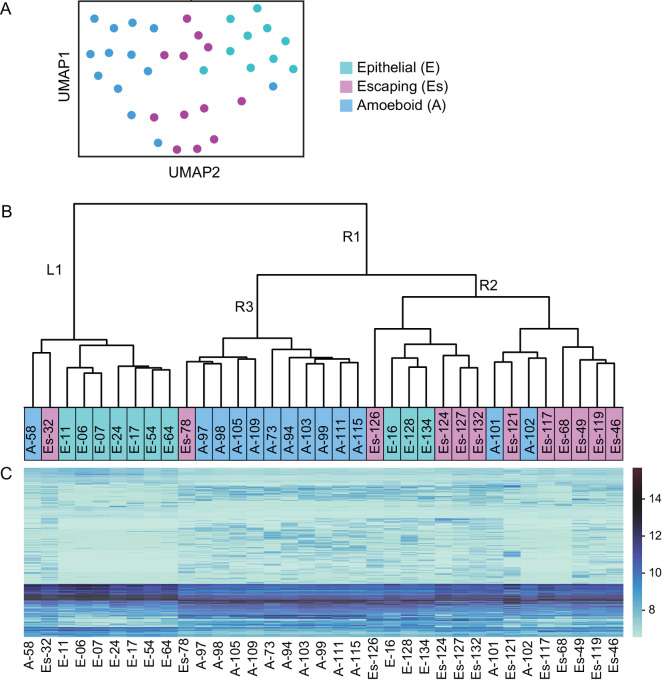
Clustering of single cells and their gene expression heat map based on 462 DE genes. **A,** UMAP clustering of 35 cells based on expression of 462 DE genes. **B,** Unsupervised hierarchical clustering of 35 cells based on the expression of 462 DE genes. Cells initially diverge into two groups, epithelial (L1) and invasive (R1), before further diverging into escaping (R2) and amoeboid (R3). **C,** Heat map of 462 DE genes for single cells arranged according to the unsupervised clustering in B.

Following elimination of outlier cells and the removal of genes with 0 detected reads across all samples, raw counts were analyzed by the DESeq2 package and VST normalization was carried out. 462 DE genes identified by DESeq2 were then used to carry out UMAP and unsupervised hierarchical clustering of the VST datasets. UMAP clustering (Fig. 3A) placed escaping cells in-between epithelial and amoeboid cells, reflecting their intermediary state. Hierarchical clustering revealed that cells broadly segregate into epithelial (L1, Fig. 3B) and invasive (R1, Fig. 3B) subsets. The invasive branch then further diverges into predominantly escaping and amoeboid subsets (R2 and R3, respectively). These clustering results confirm that the transition from an epithelial to an invasive state requires extensive transcriptional changes (26), whereas switching between invasive modes (i.e., elongated vs. rounded) is a more transient process depending chiefly on local environmental conditions (2). Imperfect clustering is not entirely surprising given the transitory nature of epithelial, escaping, and amoeboid states, the relatively small dataset, and the fact that the data were generated from a clonal cell line. The corresponding heat map for clustered cells further depicts changes in transcription between cell types (Fig. 3C).

**FIGURE 4:**
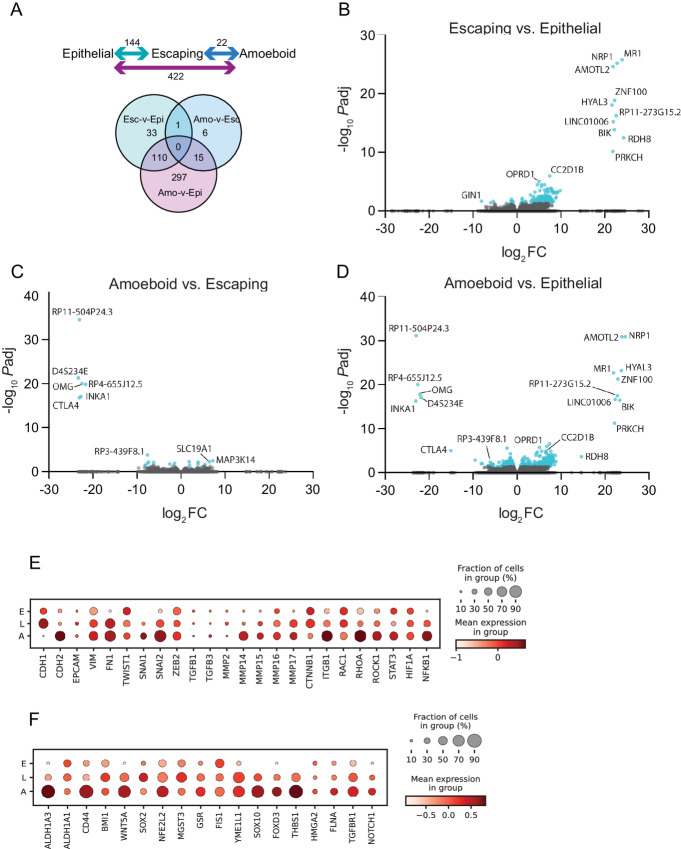
Gene expression analysis for epithelial, escaping, and amoeboid cells. **A,** Top: 462 genes were DE, 144 for escaping versus epithelial (Esc-v-Epi), 22 for amoeboid versus escaping (Amo-v-Esc), and 422 for amoeboid versus epithelial (Amo-v-Epi). Bottom: Venn diagram of the number of DE genes for the three pairwise comparisons. **B,** Volcano plot for Esc-v-Epi DE analysis, with genes enriched in “escaping” cells shown as positive on the *x*-axis. **C,** Volcano plot for Amo-v-Esc DE analysis, with genes enriched in “amoeboid” cells shown as positive on the *x*-axis. **D,** Volcano plot for Amo-v-Epi DE analysis, with genes enriched in “amoeboid” cells shown as positive on the *x*-axis. For B–D, significantly DE genes with an adjusted *P* value < 0.05 are shown in light blue, with no minimum value for log_2_FC. **E,** Expression of EMT markers for epithelial, leaving, and amoeboid cells shown via dot plot. **F,** Expression of cancer stem cell markers for epithelial, leaving, and amoeboid cells shown via dot plot.

Three comparisons were analyzed by DE analysis: escaping versus epithelial (Esc-v-Epi; reflects a partial EMT during tumor escape); amoeboid versus escaping (Amo-v-Esc; reflects MAT); and amoeboid versus epithelial (Amo-v-Epi; reflects a direct epithelial-to-amoeboid transition, or the combined effect of the two previous transitions). DE analysis identified 144, 22, and 422 DE genes (with an adjusted *P* value < 0.05) for the Esc-v-Epi, Amo-v-Esc, and Amo-v-Epi pairwise comparisons, respectively (Fig. 4A–D). The majority of DE genes identified in the Esc-v-Epi analysis were also found in the Amo-v-Epi comparison, with 33 genes specific to the Esc-v-Epi comparison. One gene, *RIMS2*, was found to be DE in both the Esc-v-Epi and Amo-v-Esc comparisons, showing increased expression specifically in escaping cells, before being downregulated upon the acquisition of an amoeboid migratory phenotype. Six genes were identified as specifically DE in the Amo-v-Esc comparison (*SLC19A1*, *CCL2*, *HOXA1*, *LPAR1*, *CCDC109B*, and lncRNA *RP3-332B11.2*), while 15 DE genes were shared between Amo-v-Esc and Amo-v-Epi comparisons. DESeq2 analysis for the Amo-v-Epi comparison generated a list of 422 DE genes, 125 of which overlapped with either Esc-v-Epi or Amo-v-Esc DE comparisons. The remaining 297 genes were unique to the Amo-v-Epi pairwise comparison. The volcano plots for the three pairwise analyses further reveal how the Amo-v-Epi comparison appears to be a combination of the Esc-v-Epi and Amo-v-Esc comparisons (Fig. 4B–D), reflecting that the transition from epithelial to an amoeboid state is indeed a three-stage process, where changes during EMT, and later MAT, are additive, and correspond to the transcriptional changes that occur between epithelial and amoeboid states. For full lists of DE genes, see Supplementary Tables S3–S5. GSEA was also carried out to identify enriched pathways using DESeq2-normalized gene expression values for 27,019 detected genes for the three comparisons described earlier (Esc-v-Epi, Amo-v-Esc, and Amo-v-Epi). Enriched pathways within the “hallmark” gene sets for each comparison are summarized in Supplementary Fig. S8 in order of significance.

Expression levels of common EMT-related genes were examined by dot plot (Fig. 4E). This identified many expected patterns such as a decrease in *CDH1* (E-cadherin), *RAC1* and *CTNNB1* (beta-catenin) as cells transition to an amoeboid state. Cells also upregulated the mesenchymal marker fibronectin upon transition from epithelial to leaving, and exhibited an increase in EMT-TFs (transcription factors) *SNAI1* and *SNAI2*, as well as amoeboid markers *RHOA* and *ROCK1*, upon transition to an amoeboid state. Cancer stem cell markers were also examined, which showed an upregulation in *ALDH1A3*, *CD44*, and *WNT5A* in amoeboid cells, and increased *SOX2* and *BMI1* in escaping cells (Fig. 4F).

Two genes whose functions in tumor escape remain elusive, *OPRD1*, encoding the delta (δ) opioid receptor, and *SLC19A1*, a folate transporter, were identified as upregulated in invasive melanoma cells and were selected for proof-of-principle perturbation to validate the predictions from the above single-cell transcriptomics and bioinformatics analyses. While it is not possible to isolate enough cells via the method described here to generate sufficient RNA or protein required for classical qPCR and Western blot analysis, respectively, SLC19A1 protein levels were analyzed in growing spheroids via immunofluorescence. This confirmed an increased level of SLC19A1 in escaped cells versus leaving cells (Supplementary Fig. S3B–S3D). Western blot analysis performed on protein isolated from cultured cells revealed widespread nonspecific binding when staining for OPRD1 (Supplementary Fig. S3A), so further immunofluorescence analysis of growing spheroids was not explored further.

### Treatment with OPRD1 Antagonist Naltrindole Prevents Melanoma Spheroid Growth and Invasion

DESeq analysis identified increased expression of *OPRD1* in both escaping and amoeboid cells when compared with epithelial cells, indicating *OPRD1* is upregulated upon tumor escape and maintained during amoeboid invasion. The *OPRD1* gene codes for the delta (δ) opioid receptor, one of the three major opioid receptors (the others being μ and κ). Opioid receptors are G protein-coupled receptors found throughout the nervous system and are most well-studied in the context of the analgesic effects that they produce upon ligand binding (27). To investigate whether OPRD1 signaling, and therefore our observation of increased *OPRD1* expression, holds a functional role in tumor escape, we carried out a 3D spheroid invasion assay in the presence of naltrindole, a selective δ-opioid receptor antagonist (28).

**FIGURE 5:**
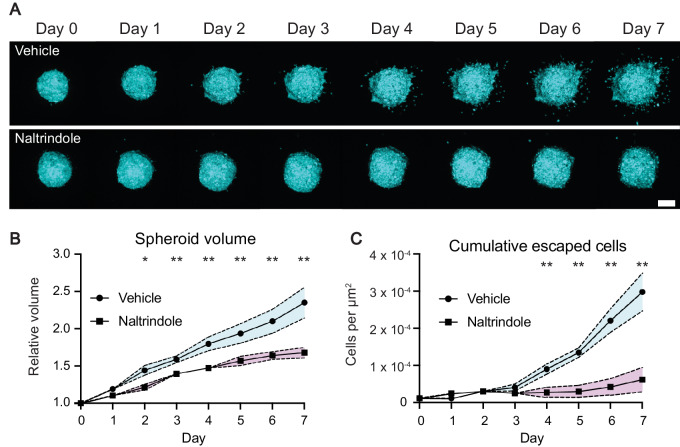
Inhibition of OPRD1 reduces melanoma spheroid growth and invasion. **A,** Representative maximum intensity projections of imaged hemispheroids for vehicle- and naltrindole-treated spheroids, scale bar 200 μm. **B,** Relative spheroid volume over time for vehicle- and naltrindole-treated cells. Shaded areas represent SEM. **C,** Cumulative number of escaped cells over time normalized to spheroid surface area. B and C, Datapoints represent the mean of eight replicates.

Spheroids were generated from WM983c-D2 cells and implanted into collagen matrix as described in Fig. 1. The following day (day 0), spheroids were imaged prior to addition of 15 μmol/L naltrindole or vehicle (nuclease-free water), and subsequently imaged daily for 7 days. Representative daily images of vehicle- and naltrindole-treated spheroids can be seen in Fig. 5A and Supplementary Videos S1A and S1B. Analysis of spheroid volume relative to day 0 revealed that naltrindole treatment induced a marked decrease in spheroid proliferation, which could be identified as early as day 2 (Fig. 5B). Strikingly, naltrindole treatment almost completely suppressed tumor escape (Fig. 5A), quantified as the number of escaped cells per spheroid over time, normalized to the surface area of the imaged hemispheroid (cells per μm^2^; Fig. 5C). For those cells that successfully escaped, there was no difference in invasion speed away from the spheroid (Supplementary Fig. S9A), but interestingly naltrindole-treated invasive cells were less rounded (Supplementary Fig. S9B). Together, these results show that naltrindole-mediated OPRD1 inhibition results in decreased spheroid growth and prevents tumor escape. The results are consistent with the increase in *OPRD1* expression identified by single-cell transcriptomics in tumor-escaping cells (Fig. 4), and suggest that the δ opioid receptor plays a critical, yet underappreciated role in the earliest phases of metastasis.

### Treatment with SLC19A1 Inhibitor SSZ Induces Tumor Escape and Elongated Invasive Melanoma Cells

**FIGURE 6:**
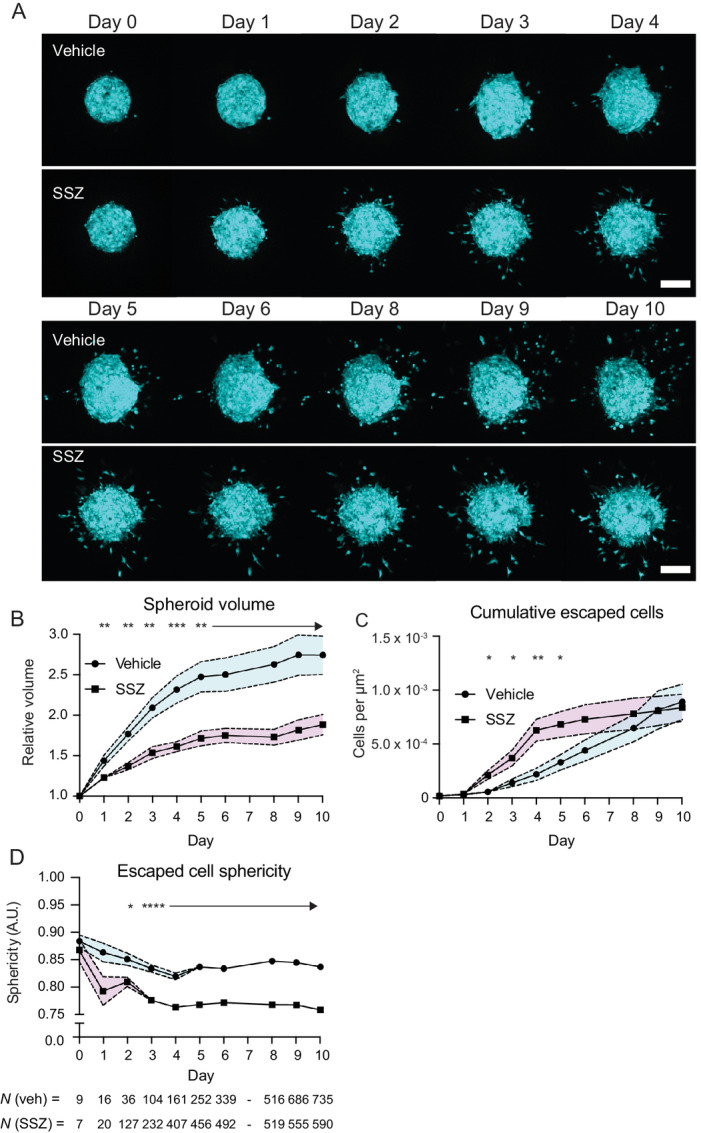
SSZ treatment slows spheroid growth but induces tumor escape by elongated cells. **A,** Representative maximum intensity projections of imaged hemispheroids for vehicle- and SSZ-treated spheroids. Scale bar, 200 μm. **B,** Relative spheroid volume for vehicle- and SSZ-treated spheroids. **C,** Cumulative escaped cells per surface area of imaged hemispheroid. **D,** Mean escaped cell sphericity per day. B–D, Shaded areas represent SEM.

The DESeq results identified a significant upregulation of the folate transporter *SLC19A1* in amoeboid cells when compared with escaping cells (Fig. 4C), suggesting that *SLC19A1* upregulation may be required for invasive amoeboid migration. To investigate this, a 3D spheroid invasion assay was performed over the course of 10 days in the presence of the SLC19A1 inhibitor, sulfasalazine (SSZ) (29). Representative images for vehicle- and SSZ-treated spheroids are shown in Fig. 6A and Supplementary Videos S2A and S2B. SSZ-treated spheroids exhibited markedly slower growth than vehicle-treated spheroids, with a difference detectable as early as day 1 (Fig. 6B). However, SSZ treatment also induced an increase in tumor escape from day 2 until day 6 (Fig. 6C). SSZ treatment did not alter the speed with which escaped cells invaded away from the spheroid (Supplementary Fig. S9C), but intriguingly, induced a marked decrease in escaped cell sphericity (Fig. 6D) consistent with the transcriptomics results, which revealed an upregulation of *SLC19A1* in amoeboid cells (Fig. 4). These data suggest that SSZ treatment, while resulting in decreased melanoma proliferation, may act as a potent inducer of tumor escape and induce a switch to mesenchymal migration in invasive cells.

## Discussion

This work describes a highly-adaptable targeted single-cell isolation method for comparative transcriptomics, applied here to identify DE genes in spontaneously-escaping melanoma cells. Coupled with light microscopy and readily accessible image analysis, this technique provides quantitative morphological and positional data up to—and immediately prior to—isolation, allowing the direct comparison of transcriptomes of individual cells of known shape and migratory state. Similar photoconvertible protein-based workflows for cell isolation and transcriptomics have been described recently (30, 31), though neither are optimized to capture specific cancer cells during the earliest stages of tumor escape, or to enable the selection of cells based on their invasive properties. Moreover, to our knowledge, there are no previous reports that isolate and compare the transcriptomes of corresponding non-invading, spontaneously escaping and amoeboid-migrating single cancer cells.

Following the capture of epithelial, escaping, and amoeboid cells, single-cell sequencing and DESeq2 analysis identified the enrichment of many genes previously implicated in tumor escape and metastasis, such as *NRP1* (32), *BIK* (33), and *PRKCH* (34) as well as some previously unstudied candidates, such as *LINC01006* and *RP11-273G15.2* (Fig. 4). Interestingly, there was widespread transcriptional upregulation upon transition from an epithelial to an escaping state, followed by widespread downregulation upon transition from an escaping to an amoeboid state. Moreover, DE genes from the Amo-v-Epi comparison largely comprised those identified in the Esc-v-Epi and Amo-v-Esc analyses, indicating that the latter transitions are additive, encompassing upregulation during tumor escape followed by downregulation during amoeboid migration. Given the pivotal role of migratory plasticity in metastasis, there have been surprisingly few published works that directly compare the transcriptomes of amoeboid versus mesenchymal tumor cells. While our “escaping” cells are largely elongated, they remain attached to the bulk spheroid following a partial EMT-like transition and can therefore not technically be termed “mesenchymal”. The “escaping” state precedes a transition to an amoeboid migratory state. Therefore, the genes identified in our single-cell Amo-v-Esc and Amo-v-Epi analyses comprise a useful resource for future research into MAT and migratory plasticity in cancer.

Because of the precise and consequently low-throughput nature of the single-cell tagging and isolation method, it is not readily achievable to collect large bulk populations of escaping and amoeboid cells, limiting suitability for qPCR-based analyses or validation of multiple individual genes. The isolation method is best paired with techniques that can extract maximal genomic, transcriptomics or epigenomic information from valuable individual cells. It is worth noting that in this instance, only 44 (29%) of cells put forward for sequencing generated mapping rates sufficient for downstream analysis (Supplementary Fig. S7F), which is lower than that reported in similar single-cell RNA-seq studies [62% (35), 84% (36), and 78% (37)]. That is, where small numbers (<1,000) of individually-processed cells are sequenced using manual, plate-based SMART-Seq technologies, in contrast to automated high-throughput droplet-based platforms such as the 10x Genomics Chromium system. Given the correlation between cDNA concentration and percentage of mapped reads per sample (Supplementary Fig. S7E), low mapping rates are likely due to low cDNA concentrations following library generation. This in turn could be either due to degradation of samples during intercontinental shipping to United States, low mRNA content prior to cDNA generation and library preparation, or issues during cDNA generation and/or library preparation such as the existence of contaminants in reagents. However, the purpose of the quality control process is precisely to identify and reject samples with insufficient quality, and we are therefore confident that the samples retained are of sufficient high quality for analysis. Furthermore, our method should achieve similar success rates as those reported above, provided single-cell cDNA library preparation is performed locally. Another consideration is the possibility for the occurrence of phototoxicity during photoconversion. In addition, all analyzed cells were exposed to the exact same photoconversion method, hence the possibility for photoconversion-induced changes in gene expression has been controlled for. However, live, Dendra2+ cells are frequently photoswitched with 405 nm lasers for RNA-seq (38, 39), often imaged for several hours or days, even *in vivo* (40, 41), so the likelihood of phototoxicity occurring during the photoconversion process is therefore minimal. In any case, the 45-minute time limit between photoconversion and cell lysis was specifically incorporated to minimize the effects of altered gene expression caused by experimental handing, including photoconversion.

Changes in EMT signature gene expression across cell types were largely as expected, for example, the “cadherin switch” is observed during the transition from escaping to amoeboid, with a downregulation of E-cadherin (*CDH1*) and beta-catenin (*CTNNB1*), and upregulation of N-cadherin (*CDH2*; Fig. 4D). Despite being in an epithelial-like state at the spheroid edge, the absence of *EPCAM* is not surprising due to the melanocytic origins of the cell line. Both EMT-TFs *SNAI1* and *SNAI2* increased as cells transitioned from epithelial to escaping and amoeboid states, though *TWIST1* and *ZEB2* were unchanged. This is consistent with a previous report, which identified inconsistent EMT-TF expression between wildtype and migratory cells of various breast cancer cell lines (42). Our observed decrease in *RAC1* and increase in *RHOA* and *ROCK1* are hallmarks of contractile amoeboid migration (Fig. 4D). While amoeboid cells are usually associated with decreased expression of proteases, we observed no change in *MMP2*, *15*, *16*, *17*, and an upregulation of *MMP14* upon amoeboid transition (Fig. 4D). Increasing studies are identifying “uncharacteristic” protease expression in amoeboid cells (43), further highlighting the heterogeneity of the amoeboid state. We also observed an upregulation of migratory neural crest genes *SOX10* and *FOXD3* (Fig. 4E; ref. 44) in escaping and amoeboid cells. This is not surprising given that melanocytes differentiate from neural crest cells (45), and the shared gene signatures between developmental (type I) and pathophysiologic (type III) EMT programmes (46). A recent study examined gene expression in keratinocytes and identified a migratory *THBS1*+ subpopulation induced upon wound healing (47). Interestingly, we also observe an increased expression of *THBS1* in escaping and amoeboid cells (Fig. 4E), likely via increased RHO/ROCK signaling (47), which follows a similar trend in our data set. Observed differences in transcriptional profiles were not due to batch-batch or day-day variation, as we ensured to capture a range of “ages” for each cell type (epithelial, leaving, amoeboid; Supplementary Table S1), and cells included in the final analysis were from a range of sorting events (Supplementary Table S6).

Two candidate genes identified by the comparative single-cell transcriptomics reported here were further investigated, in part to test the sensitivity and potential for discovery of our method. Strikingly, pharmacologic inhibition of both OPRD1 and SLC19A1 induced changes in invasion and migratory mode consistent with what was predicted by our DE analysis. The DE analysis identified an upregulation of *OPRD1* in both escaping and amoeboid cells when compared with epithelial cells (Fig. 4B and D), indicating that the δ opioid receptor supports tumor escape. Employing the same invasion assay established earlier in the study, the effect of OPRD1 inhibition was investigated with the use of the selective OPRD1 antagonist naltrindole (28). Naltrindole treatment resulted in a stark reduction in both spheroid growth and invasion (Fig. 5), indicating that δ opioid receptor signaling plays a critical role in tumor escape, as well as tumor proliferation. Previous studies have identified *OPRD1* as being upregulated in cancer (48) and use of opioid-based analgesia during cancer treatment has been correlated with increased metastasis and decreased survival (49–53), though the mechanisms underpinning the role of *OPRD1* in metastasis are largely unknown. However, a recent study (54) identified a role for OPRD1 signaling in JAK1/2-STAT3-mediated metastasis, which was abolished by the addition of naltrindole. Interestingly, an enrichment of JAK1/2-STAT3 signaling in amoeboid cells was also identified during GSEA of our data, which may well be a consequence of increased OPRD1 signaling (Supplementary Fig. S8).

The comparative single-cell transcriptomics also identified an upregulation of folate transporter *SLC19A1* in amoeboid cells when compared with escaping cells (Fig. 4C), implicating SLC19A1 activity in rounded amoeboid invasion. SLC19A1 encodes a reduced folate carrier that acts as an antiporter, coupling export of organic phosphates to the import of folate (55). It has also more recently been identified as a major transporter of extracellular cyclic dinucleotides into the cell to induce the proinflammatory stimulator of interferon genes (STING) pathway (55, 56). While increased folate metabolism in cancer cells is a well-reported phenomenon, and the use of antifolates was one of the earliest forms of leukemia chemotherapy (57), the role of SLC19A1 is largely unstudied in the context of tumor escape and invasion. To investigate the effects of SLC19A1 inhibition in this context, an invasion assay following the addition of sulfasalazine (SSZ), a potent non-competitive inhibitor of SLC19A1 (29), was conducted. This indicated that SSZ treatment decreased spheroid growth (Fig. 6A and B), instead inducing tumor escape (Fig. 6C). The results suggest that SSZ treatment of solid tumors causes cells at the periphery of the growing mass to switch to an invasive, elongated phenotype, consistent with EMT. Furthermore, consistent with the observation of *SLC19A1* upregulation in amoeboid cells, SSZ treatment caused invasive cells to adopt an elongated shape (Fig. 6D) without loss of invasion speed (Supplementary Fig. S9C). This further suggests that a reduction in folate uptake by melanoma tumor cells induces EMT, and an elongated migratory phenotype consistent with mesenchymally-migrating tumor cells. Interestingly, SSZ has been suggested as a potential therapeutic agent for cancer patients. This is based on its well-studied anti-inflammatory effects and ability to induce chemosensitivity (58, 59). Furthermore, SSZ is currently being explored therapeutically as a treatment in patients with cancer, though this is based on its ability to reduce pain via efflux of glutamate due to its inhibitory effect on the cysteine/glutamate antiporter SLC7A11 (Clinical Trial NCT number NCT03847311). In our experiments, SSZ treatment caused a rapid and robust induction in tumor escape (Fig. 6C), which after 5 days slowed to return to baseline levels, likely due to decay, cellular uptake, and metabolism of the drug, which could be mitigated by periodic replenishment with fresh SSZ. This contrasts with the elongated shapes of invading cells sustained for the duration of the experiment (Fig. 6D), which indicates that the elongated invasive migration mode induced by SSZ during tumor escape is a result of a permanent or longer-term cellular reprogramming. In other words, if the elongated migratory phenotype was reversible, we would expect to see the return of more rounded escaped cells as extracellular SSZ concentrations diminish. Therefore, and given the use of nonlimiting concentrations of SSZ in the mmol/L range, it is tempting to speculate that the marked change in tumor escape around day 5 is driven by more complex factors than merely diminishing exposure to the drug. Future pharmacokinetics studies of SSZ stability and metabolism by cancer cells, beyond the scope of this work, will shed further light on how transient its induction of tumor escape is.

While the importance of *in vivo* models for the study of cancer cannot be understated, especially given the highly complex and diverse effects of the heterogeneous tumor microenvironment (60), the study of events such as EMT and MAT in a controlled setting such as described here should not be overlooked. The single-cell isolation method described herein opens the door to the identification of fundamental transcriptomics changes underpinning tumor escape and invasion. Moreover, its simplicity, adaptability, and precision make it a valuable and accessible tool for the provision of targeted single cells from a wide range of pathologic and homeostatic models where comparative single-cell data can help resolve hitherto intractable questions.

## Supplementary Material

Supplementary Figure 1 Supplementary Figure 1 shows data generation for the photoconvertible spheroid assay

Supplementary Figure 2 Supplementary Figure 2 shows clustering of 44 single cells and their gene expression heat map based on 230 differentially expressed genes

Supplementary Figure 3 Supplementary Figure 3 shows validation of OPRD1 and SLC19A1 protein expression

Supplementary Figure 4 Supplementary Figure 4 shows an Epithelial cohort gallery

Supplementary Figure 5 Supplementary Figure 5 shows an Escaping cell cohort gallery

Supplementary Figure 6 Supplementary Figure 6 shows an Amoeboid cell cohort gallery

Supplementary Figure 7 Supplementary Figure 7 shows metadata for isolated cells

Supplementary Figure 8 Supplementary Figure 8 shows a summary of GSEA pathway analyses using Hallmarks gene set showing top 25 enriched pathways for each comparison

Supplementary Figure 9 Supplementary Figure 9 shows Escaped cell analyses for naltrindole and sulfasalazine spheroid assays

Supplementary Table 1 Supplementary Table 1 shows Cell type isolated, age (days) and number of cells sorted for each spheroid included in the study

Supplementary Table 2 Supplementary Table 2 shows Transcript counts for common housekeeping genes, with cells excluded from the study due to low housekeeping expression highlighted in yellow

Supplementary Table 3 Supplementary Table 3 shows Differentially expressed genes for escaping vs. epithelial analysis

Supplementary Table 4 Supplementary Table 4 shows Differentially expressed genes for amoeboid vs. escaping analysis

Supplementary Table 5 Supplementary Table 5 shows Differentially expressed genes for epithelial vs. amoeboid analysis

Supplementary Table 6 Supplementary Table 6 shows Sorting events for each cell included in the final data set

Supplementary Video 1A Supplementary Video 1A shows representative daily images of a vehicle-treated spheroid. Scale bar: 200 µm, number at top left: days post-implantation.

Supplementary Video 1B Supplementary Video 1B shows representative images of a naltrindole-treated spheroid. Scale bar: 200 µm, number at top left: days post-implantation.

Supplementary Video 2A Supplementary Video 2A shows representative images of a vehicle-treated spheroid. Scale bar: 200 µm, number at top left: days post-implantation.

Supplementary Video 2B Supplementary Video 2B shows representative images of a Sulfasalazine-treated spheroid. Scale bar: 200 µm, number at top left: days post-implantation.
