# Supplementary material for: Targeted Single-cell Isolation of Spontaneously Escaping Live Melanoma Cells for Comparative Transcriptomics
Source: Cancer Res Commun. 2023 Aug 11;3(8):1524–37. doi: 10.1158/2767-9764.CRC-22-0305 (PMC10416804; doi:10.1158/2767-9764.CRC-22-0305)
Supplement: Supplementary Figure 1 — shows data generation for the photoconvertible spheroid assay [file crc-22-0305-s01.pdf]

Supplementary Figure 1

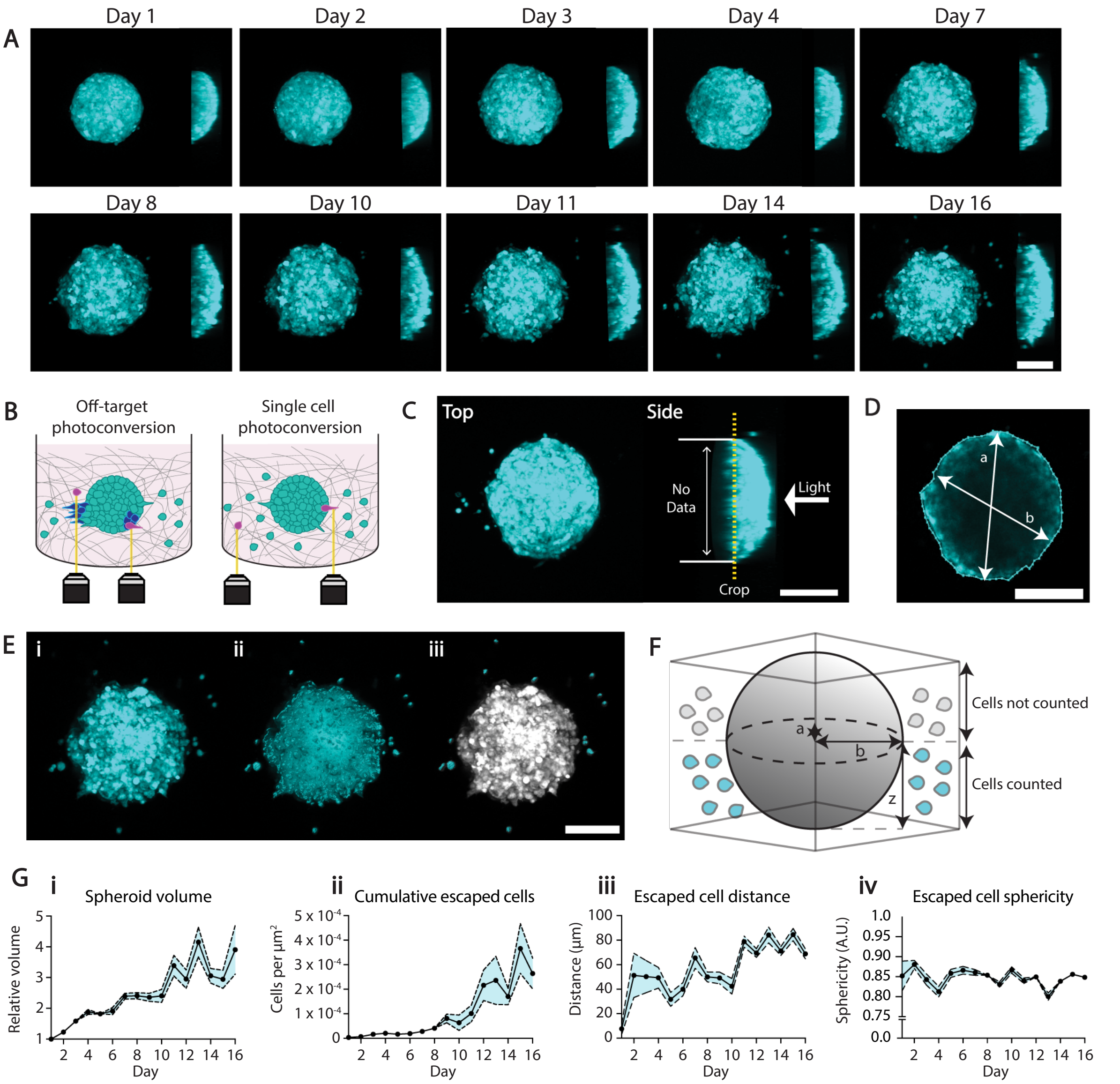

H

Day	1	2	3	4	5	6	7	8	9	10	11	12	13	14	15	16
$N_{total\ spheroids}$	46	33	19	28	17	13	23	34	14	8	18	12	4	15	8	8
$N_{invasive\ spheroids}$	8	10	11	16	10	8	16	27	14	6	15	12	4	14	8	8
$N_{escaped\ cells}$	8	14	24	40	31	30	63	152	128	49	162	270	114	280	366	323

Supplementary Figure 1 | Data generation for the photoconvertible spheroid assay All scale bars show 200 µm. **A** | Representative top and side view maximum intensity projections of a 3D melanoma spheroid cultured over 16 days post implantation. Spheroids increase in volume over time and rounded escaped cells are visible from day 7. **B** | Off-target photoconversion (shown in dark blue) occurs when cells other than the target cell (pink) are photoconverted in the path of the laser beam. For correct single cell photoconversion using single photon light, only target cells with no other cells above or below the laser can be safely photoconverted. **C** | Top and side view of imaged spheroid. Due to the limitations of confocal imaging, it is not possible to obtain fluorescence data from behind the spheroid, so 3D images were cropped at the widest point, as shown by the yellow dotted line. **D** | An automatic isoline was used to generate a flat surface, and ellipsoid axes a and b were provided by the Imaris software. **E** | Fluorescence (i) data was used to perform surface rendering of entire 3D image (ii) and surfaces corresponding to the bulk spheroid were manually removed (iii). **F** | Only cells within 'z' were counted for analysis due to cropping of 3D images, and the isoline generated in D was used to provide 'a' and 'b' values. **G** | Spheroid growth and invasion analyses. i) Mean daily spheroid volume normalised to day 1 volume, N = Ntotal spheroids. ii) total number of escaped cells on a given day per µm2 surface area of spheroid per day, N = Ntotal spheroids. iii) mean distance of escaped cells from spheroid edge per day, N = Nescaped cells. iv) Mean escaped cell sphericity per day, N = Nescaped cells. N numbers are denoted in **H**. Note that Ntotal spheroids is not constant due to samples being imaged every 1-3 days, and sample numbers reducing over time as spheroids were tagged and processed for sequencing.
