# Supplementary material for: Targeted Single-cell Isolation of Spontaneously Escaping Live Melanoma Cells for Comparative Transcriptomics
Source: Cancer Res Commun. 2023 Aug 11;3(8):1524–37. doi: 10.1158/2767-9764.CRC-22-0305 (PMC10416804; doi:10.1158/2767-9764.CRC-22-0305)
Supplement: Supplementary Figure 2 — shows clustering of 44 single cells and their gene expression heat map based on 230 differentially expressed genes [file crc-22-0305-s02.pdf]

Supplementary Figure 2

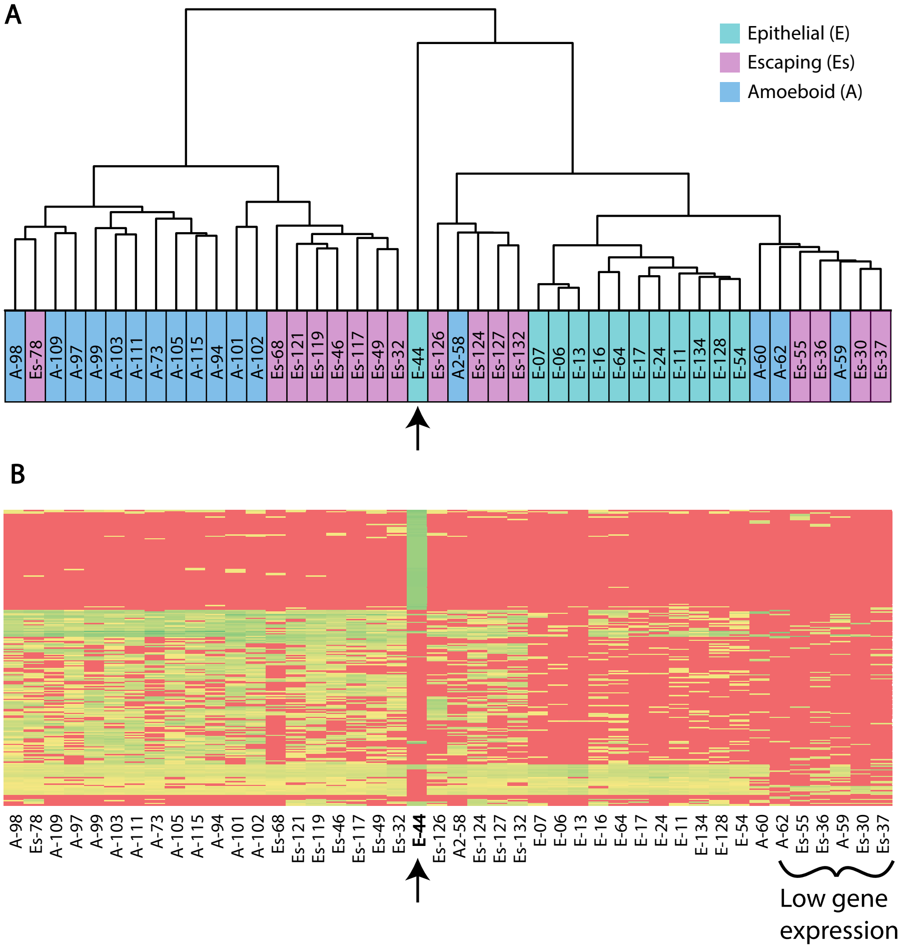

Supplementary Figure 2 | Clustering of 44 single cells and their gene expression heat map based on 230 differentially expressed genes

A | Unsupervised hierarchical clustering of 44 cells based on the expression of 230 DE genes identified using DESeq2. **B** | Heat map of 230 DE genes for single cells arranged according to the unsupervised clustering in A.
