# Supplementary material for: Targeted Single-cell Isolation of Spontaneously Escaping Live Melanoma Cells for Comparative Transcriptomics
Source: Cancer Res Commun. 2023 Aug 11;3(8):1524–37. doi: 10.1158/2767-9764.CRC-22-0305 (PMC10416804; doi:10.1158/2767-9764.CRC-22-0305)
Supplement: Supplementary Figure 3 — shows validation of OPRD1 and SLC19A1 protein expression [file crc-22-0305-s03.pdf]

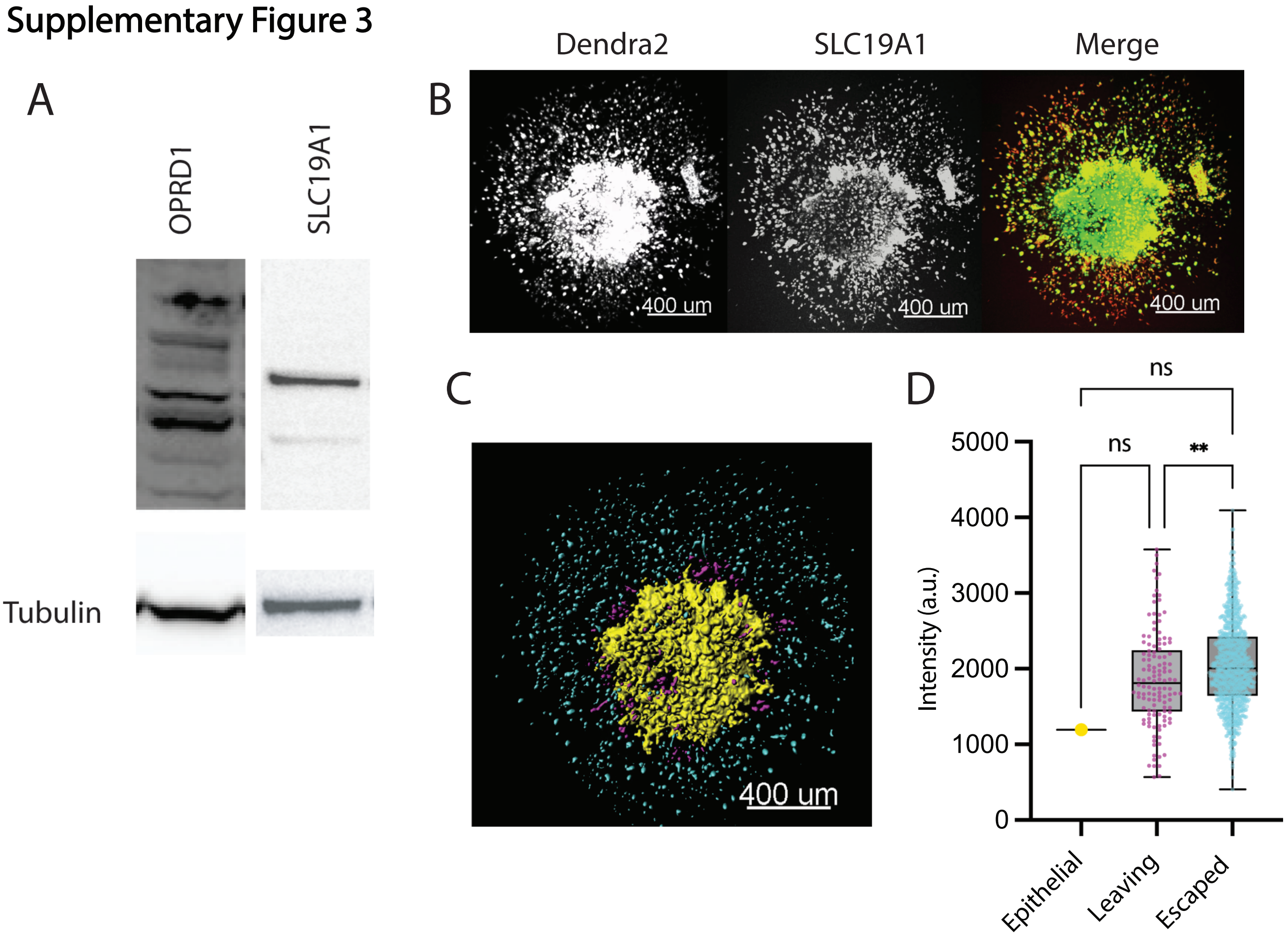

Supplementary Figure 3 | Validation of OPRD1 and SLC19A1 protein expression. **A** | Western blot analyses for OPRD1 and SLC19A1 in WM983c G1/M1 cell line. **B** | Dendra2 fluorescence and SLC19A1 immunofluorescence in growing WM983c-G1/M1 spheroids. In merge, Dendra2 is shown in green and SLC19A1 in red. **C** |Surfaces for epithelial (yellow) leaving (magenta) and escaped (cyan) cells used for quantification of fluorescence intensity in the SLC19A1 channel. **D** |Box plot depicting the mean fluorescence intensity for surfaces shown in C. P-values are via Kruskal-Wallis nonparametric test followed by Dunn's multiple comparison. P-value for leaving vs. escaped is 0.0013.
