# Supplementary material for: Targeted Single-cell Isolation of Spontaneously Escaping Live Melanoma Cells for Comparative Transcriptomics
Source: Cancer Res Commun. 2023 Aug 11;3(8):1524–37. doi: 10.1158/2767-9764.CRC-22-0305 (PMC10416804; doi:10.1158/2767-9764.CRC-22-0305)
Supplement: Supplementary Figure 4 — shows an Epithelial cohort gallery [file crc-22-0305-s04.pdf]

Supplementary Figure 4

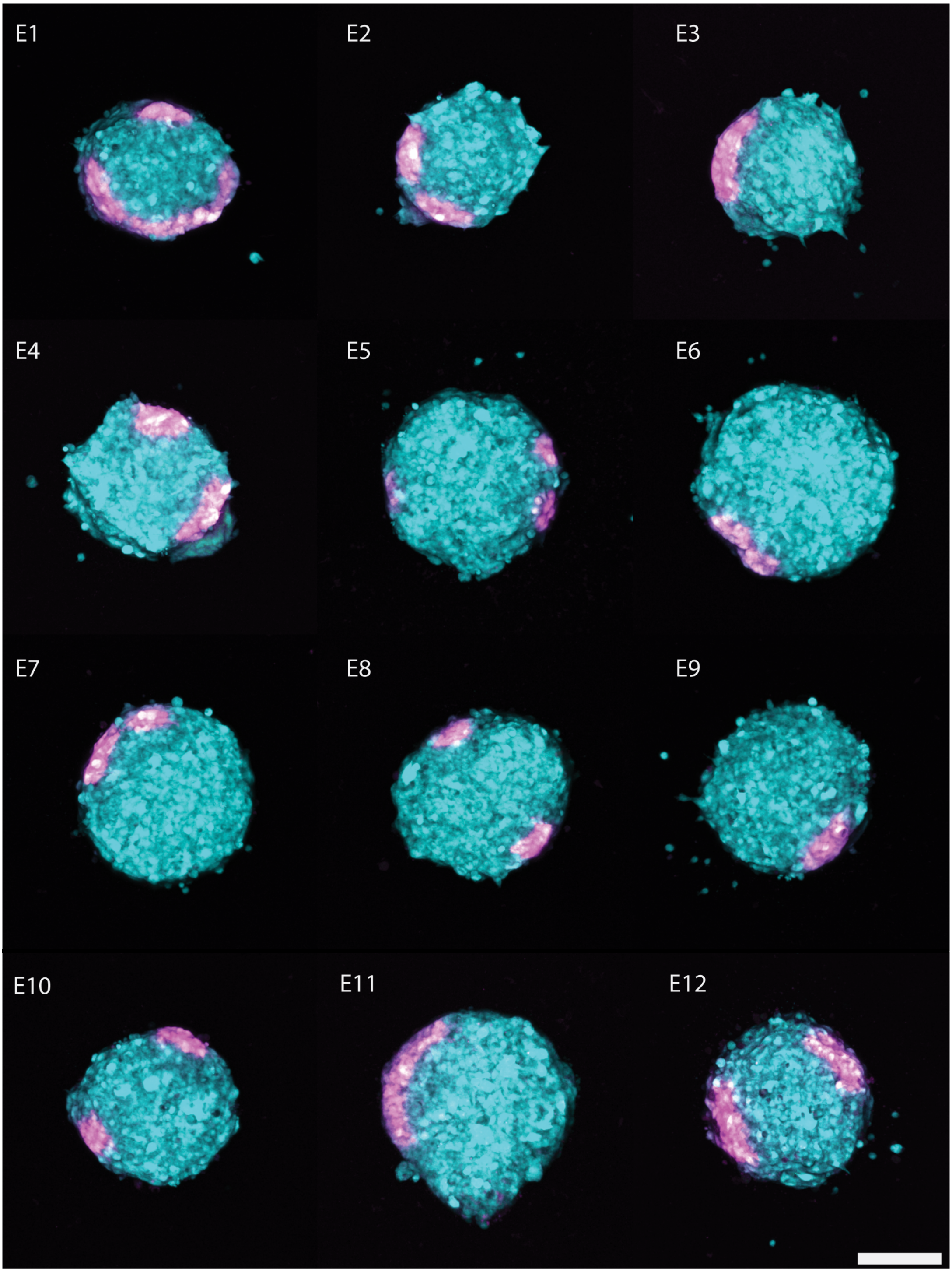

Supplementary Figure 4 | Epithelial cohort gallery. Epithelial cells were photoconverted at the edge of the spheroid from a total of 12 spheroid samples. Photoconverted cells are shown in magenta. Scale bar 200 μm.
