# Supplementary material for: Targeted Single-cell Isolation of Spontaneously Escaping Live Melanoma Cells for Comparative Transcriptomics
Source: Cancer Res Commun. 2023 Aug 11;3(8):1524–37. doi: 10.1158/2767-9764.CRC-22-0305 (PMC10416804; doi:10.1158/2767-9764.CRC-22-0305)
Supplement: Supplementary Figure 5 — shows an Escaping cell cohort gallery [file crc-22-0305-s05.pdf]

Supplementary Figure 5

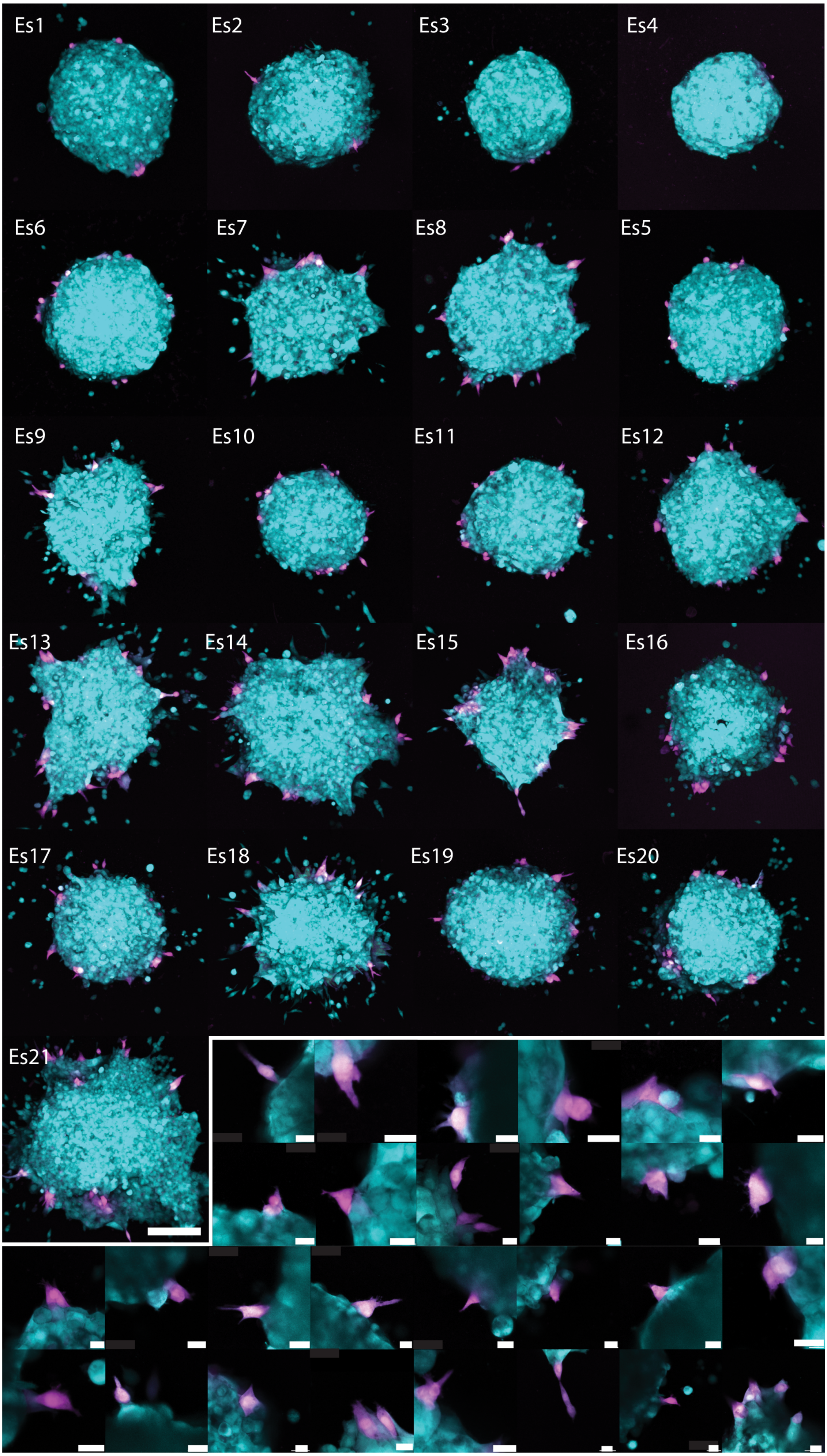

Supplementary Figure 5 | Escaping cell cohort gallery. Invasive escaping cells still attached to the spheroid were photoconverted from a total of 21 spheroid samples. Photoconverted cells are shown in magenta. Scale bar 200 µm for whole field of view images, 20 µm for close-ups.
