# Supplementary material for: Targeted Single-cell Isolation of Spontaneously Escaping Live Melanoma Cells for Comparative Transcriptomics
Source: Cancer Res Commun. 2023 Aug 11;3(8):1524–37. doi: 10.1158/2767-9764.CRC-22-0305 (PMC10416804; doi:10.1158/2767-9764.CRC-22-0305)
Supplement: Supplementary Figure 6 — shows an Amoeboid cell cohort gallery [file crc-22-0305-s06.pdf]

Supplementary Figure 6

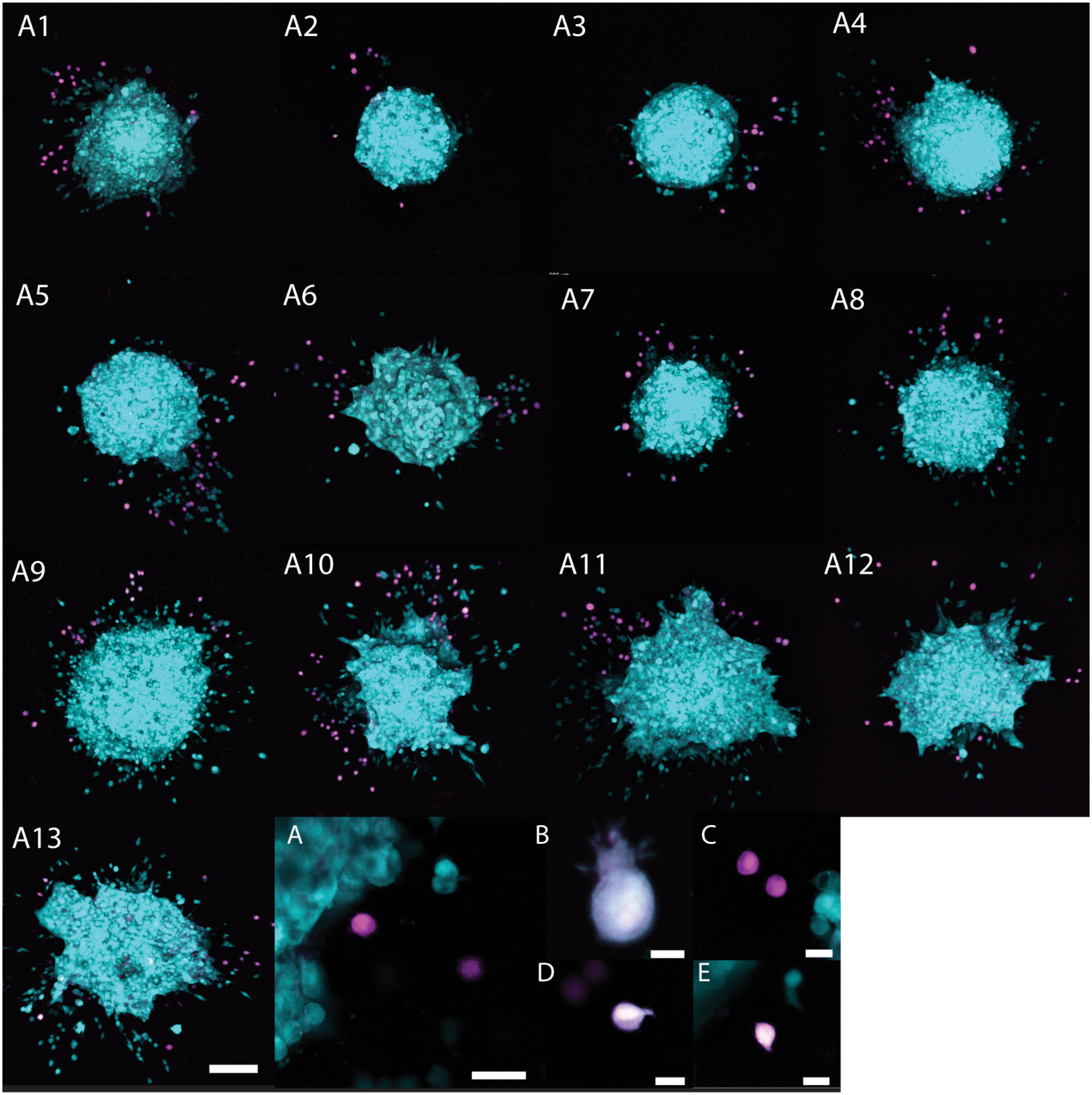

Supplementary Figure 6 | Amoeboid cell cohort gallery. Rounded amoeboid cells were photo-converted from 13 spheroid samples. Photoconverted cells are shown in magenta. Scale bar: 200 µm for whole field of view; for A: 30 µm; B: 10 µm; C-E: 20 µm.
