# Supplementary material for: Targeted Single-cell Isolation of Spontaneously Escaping Live Melanoma Cells for Comparative Transcriptomics
Source: Cancer Res Commun. 2023 Aug 11;3(8):1524–37. doi: 10.1158/2767-9764.CRC-22-0305 (PMC10416804; doi:10.1158/2767-9764.CRC-22-0305)
Supplement: Supplementary Figure 7 — shows metadata for isolated cells [file crc-22-0305-s07.pdf]

Supplementary Figure 7

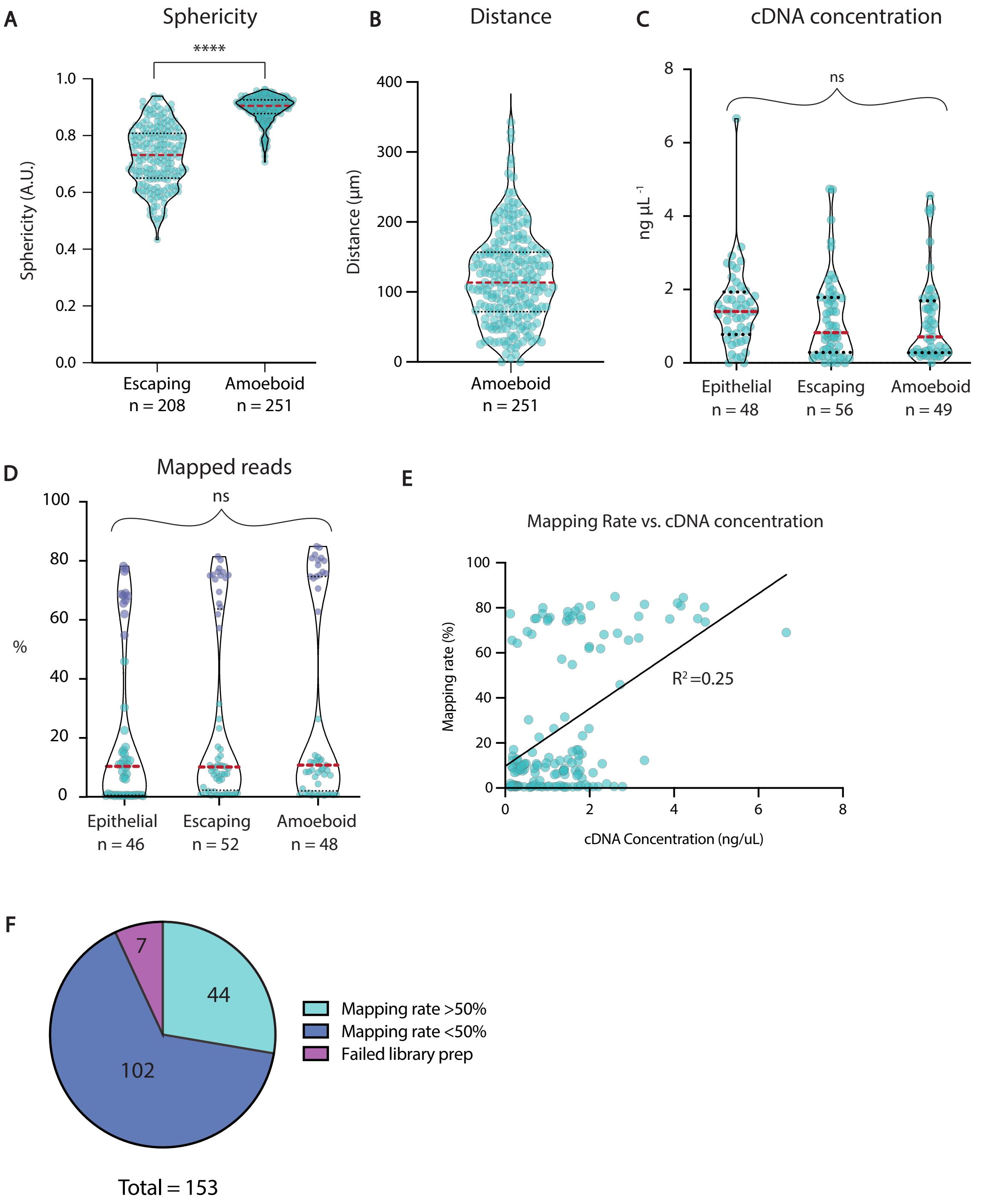

Supplementary Figure 7 | Metadata for isolated cells. Each dot represents a single cell. **A** | Sphericity values for photoconverted escaping and amoeboid cells. **B** | Distance from spheroid edge for photoconverted amoeboid cells. **C** | cDNA concentrations for epithelial, escaping and amoeboid samples. **D** | Percentage of reads successfully mapped to the human reference genome for each cell sample. Samples with mapping rates >50% were retained for analysis, shown in purple. cDNA concentrations and mapped reads were found not to be significantly different via Kruskal-Wallis H test. **E** | Scatter plot of mapping rate (%) vs. cDNA concentration (ng µL-1). Line generated by simple linear regression. **F** | Pie chart depicting the outcomes of the 153 cells processed for scRNA-seq.
