# Supplementary material for: Targeted Single-cell Isolation of Spontaneously Escaping Live Melanoma Cells for Comparative Transcriptomics
Source: Cancer Res Commun. 2023 Aug 11;3(8):1524–37. doi: 10.1158/2767-9764.CRC-22-0305 (PMC10416804; doi:10.1158/2767-9764.CRC-22-0305)
Supplement: Supplementary Figure 8 — shows a summary of GSEA pathway analyses using Hallmarks gene set showing top 25 enriched pathways for each comparison [file crc-22-0305-s08.pdf]

Supplementary Figure 8

Epithelial ↔ Escaping

Epithelial	Escaping
Oxidative phosphorylation	**Mitotic spindle**
	Glycolysis
	**G2M checkpoint**
	IFNγ reponse
	**Hedgehog signaling**
	IFNα response
	Hypoxia
	**E2F targets**
	MTORC1 signaling
	UV response (up)
	Pancreas β cells
	TGFβ signaling
	IL2 STAT5 signaling
	**KRAS signaling (down)**
	Complement
	Fatty acid metabolism
	**Bile acid metabolism**
	UV response (down)
	Inflammatory response
	Spermatogenesis
	Coagulation
	Notch signaling
	Adipogenesis
	EMT
	Apoptosis

Escaping ↔ Amoeboid

Escaping	Amoeboid
**KRAS signaling (down)**	**Myc targets (v2)**
KRAS signaling (up)	**EMT**
**Mitotic spindle**	**Oxidative phosphorylation**
**G2M checkpoint**	**Adipogenesis**
**Hedgehog signaling**	**Myc targets (v1)**
Estrogen response early	**Apoptosis**
**Bile acid metabolism**	**MTORC1 signaling**
WNT β catenin signaling	**Unfolded protein response**
**E2F targets**	**Fatty acid metabolism**
	Protein secretion
	Allograft rejection
	**Coagulation**
	**PI3K AKT MTOR signaling**
	ROS pathway
	**Apical junction**
	**TGFβ signaling**
	DNA repair
	**Xenobiotic metabolism**
	**Glycolysis**
	**Androgen response**
	**Apical surface**
	Peroxisome
	IL6 JAK STAT3 signaling
	Myogenesis
	**IFNγ reponse**

Epithelial ↔ Amoeboid

Epithelial	Amoeboid	
KRAS signaling (down)	**Glycolysis**	**Coagulation**
	**MTORC signaling**	**Apical junction**
	**MYC targets (v1)**	Hedgehog signaling
	**Adipogenesis**	Complement
	**Fatty acid metabolism**	**PI3K AKT MTOR signaling**
	**Myc targets (v2)**	Hypoxia
	**EMT**	UV response (up)
	**Unfolded protein response**	IFNα response
	**IFNγ response**	**Apical surface**
	**TGFβ signaling**	**Apoptosis**
	**Androgen response**	Spermatogenesis
	**Xenobiotic metabolism**	UV reponse (down)
	**Oxidative phosphorylation**	

Supplementary Figure 8 | Summary of GSEA pathway analyses using Hallmarks gene set showing top 25 enriched pathways for each comparison. Pathways highlighted in bold appear in the top 25 enriched pathways of the same cell type in alternate comparisons.
