# Supplementary material for: Targeted Single-cell Isolation of Spontaneously Escaping Live Melanoma Cells for Comparative Transcriptomics
Source: Cancer Res Commun. 2023 Aug 11;3(8):1524–37. doi: 10.1158/2767-9764.CRC-22-0305 (PMC10416804; doi:10.1158/2767-9764.CRC-22-0305)
Supplement: Supplementary Figure 9 — shows Escaped cell analyses for naltrindole and sulfasalazine spheroid assays [file crc-22-0305-s09.pdf]

Supplementary Figure 9

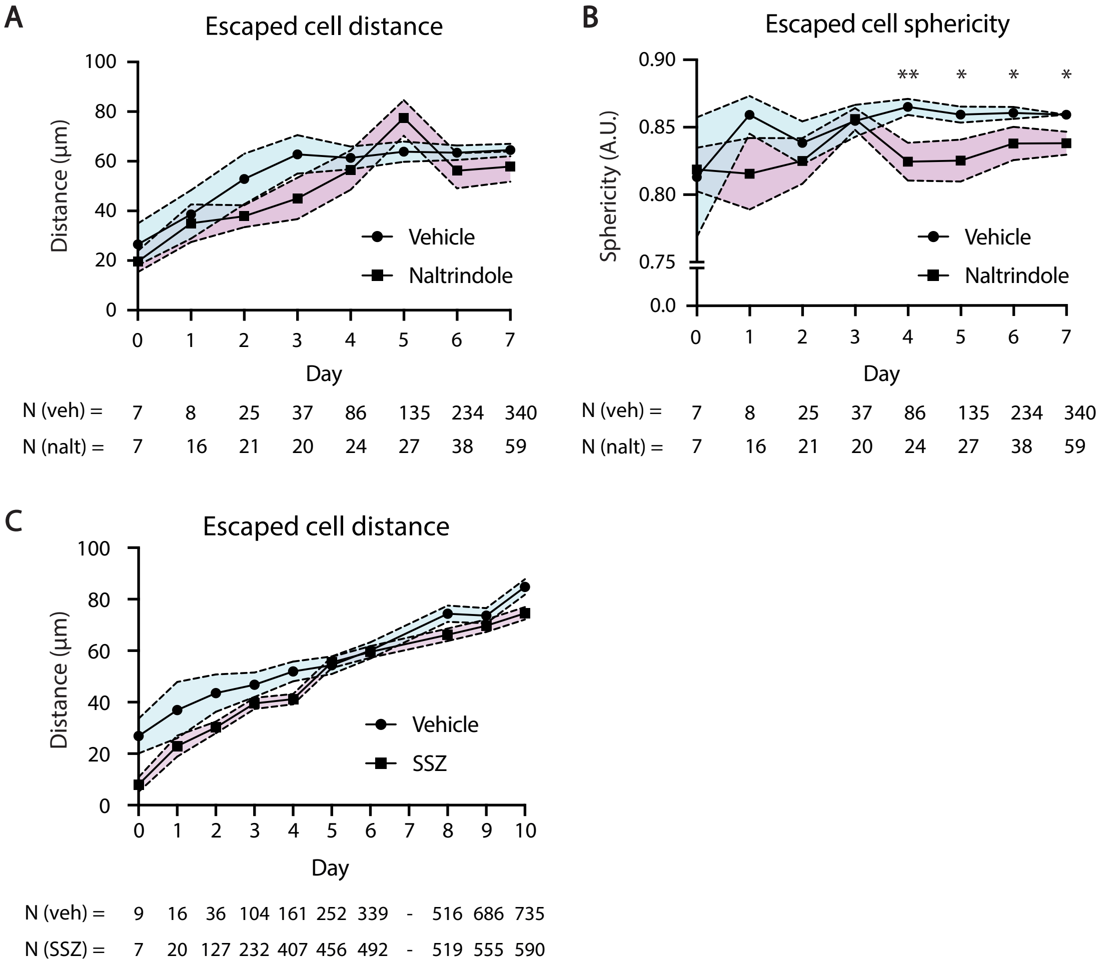

Supplementary Figure 9 | Escaped cell analyses for naltrindole and sulfasalazine spheroid assays. **A** | Mean escaped cell distance from spheroid edge for vehicle and naltrindole treated spheroids. **B** | Mean escaped cell sphericity for vehicle and naltrindole treated spheroids. **C** | Mean escaped cell distance for vehicle and sulfasalazine treated spheroids.
